# Key needs, quality performance indicators and outcomes for patients with atrial fibrillation and multimorbidity: The AFFIRMO study

**DOI:** 10.1371/journal.pone.0310106

**Published:** 2024-09-10

**Authors:** Donato Giuseppe Leo, Caterina Trevisan, Adele Ravelli, Trudie C. A. Lobban, Deirdre A. Lane, on behalf of the AFFIRMO Study investigators

**Affiliations:** 1 Liverpool Centre for Cardiovascular Sciences, University of Liverpool and Liverpool Heart and Chest Hospital, Liverpool, United Kingdom; 2 Department of Cardiovascular and Metabolic Medicine, Faculty of Health and Life Sciences, Institute of Life Course and Medical Sciences, University of Liverpool, Liverpool, United Kingdom; 3 Department of Medicine, University of Padua, Padua, Italy; 4 Department of Medical Sciences, University of Ferrara, Ferrara, Italy; 5 Aging Research Center, Department of Neurobiology, Care Sciences and Society (NVS), Karolinska Institutet-Stockholm University, Stockholm, Sweden; 6 Arrhythmia Alliance, Stratford upon Avon, Warwickshire, United Kingdom; 7 Department of Clinical Medicine, Aalborg University, Aalborg, Denmark; Cook County Health, UNITED STATES OF AMERICA

## Abstract

**Background:**

Patients with atrial fibrillation (AF) often have concomitant long-term conditions that negatively impact their quality of life and the clinical management they receive. The AFFIRMO study aimed to identify the needs, quality performance indicators (QPIs), and outcomes relevant to patients, caregivers and healthcare professionals (HCPs) to improve the care of patients with AF.

**Methods:**

An on-line survey to collect the key needs, QPIs, and outcomes relevant to patients with AF, their caregivers and HCPs, was distributed between May 2022 and January 2023 in five countries (UK, Italy, Denmark, Romania and Spain). Results from the on-line survey were discussed in a three-round Delphi process with international representatives of patients with AF, caregivers, and HCPs to determine the key needs, QPIs and outcomes for the management of patients with AF and multimorbidity.

**Results:**

659 patients (47.2% males, mean (SD) age 70.9 (10.2) years), 201 caregivers (26.9% males, mean (SD) age: 58.3 (SD 15.2) years), and 445 HCPs (57.8% males, mean (SD) age 47.4 (10.6) years) participated in the survey. An initial list of 27 needs, 9 QPIs, and 17 outcomes were identified. Eight patients, two caregivers, and 11 HCPs participated in the Delphi process. Nineteen (70%) needs, 8 (89%) QPIs, and 13 (76%) outcomes reached “consensus in”, and were included in the final list.

**Conclusions:**

The final key needs, QPIs and outcomes obtained from the Delphi process will inform the AFFIRMO clinical trial, which aims to test the iABC app which incorporates an empowerment toolbox for patients and their caregivers, providing information to improve patient engagement and empowerment to help improve the clinical and self-management of patients with AF in the context of multimorbidity.

## Introduction

Atrial fibrillation (AF) affects 1–2% of the European population [1], with its incidence increasing particularly among people aged 65 years and older [1]. In recent years, the management of AF has embraced a more integrated care approach in the form of the ABC (Atrial Fibrillation Better Care) pathway [2–4], however there is still a large discrepancy in the management of patients within and between countries [4, 5].

Multimorbidity, defined as the concomitant presence of two or more chronic health conditions [6, 7], is very common in patients with AF, who often report a higher multimorbidity rate compared to the non-AF population [8, 9]. The most common comorbidities among patients with AF are hypertension, heart failure and chronic kidney disease, all of which are associated with a higher risk of hospitalisation, and all-cause mortality [9]. The burden of multimorbidity worsens with ageing, negatively affecting the patients’ quality of life, and increasing the complexity of their clinical management [6, 7].

Ignoring the heterogeneous spectrum of disease combinations typical of multimorbidity, the majority of the European healthcare systems still adopt a single-disease approach, which reduces the likelihood of a holistic approach, taking into account the overall health of the patient [10]. A more patient-centred approach is advocated in order to address the needs of patients with AF in the context of multimorbidity [11, 12], also considering key factors such as increased patient education and health literacy [13], and assessment of quality performance indicators (QPIs) of care [14]. Moreover, it is crucial empowering patients to be more involved in the management of their health and to become partners with the healthcare team in the decision-making process concerning their care [15, 16].

The Atrial Fibrillation Integrated Approach in Frail, Multimorbid, and Polymedicated Older People (AFFIRMO) project [17] aims to improve the management of AF in the context of multimorbidity, with the focus on a holistic approach to optimise clinical management of older patients with AF, considering the multifaceted aspects of individuals’ health, including multimorbidity, polypharmacy, personal preferences, and social context. The AFFIRMO sub-study reported here aimed to identify the key needs, QPIs, and outcomes relevant to patients with AF and multimorbidity, their caregivers, and healthcare professionals (HCP) involved in their clinical management. This aim was to understand the key aspects of care that need to be improved in the management of these patients and to inform the clinical trial of the AFFIRMO study, where an empowerment toolbox will be provided to patients and caregivers, enabling them to receive information about their health conditions.

## Methods

**Fig 1:**
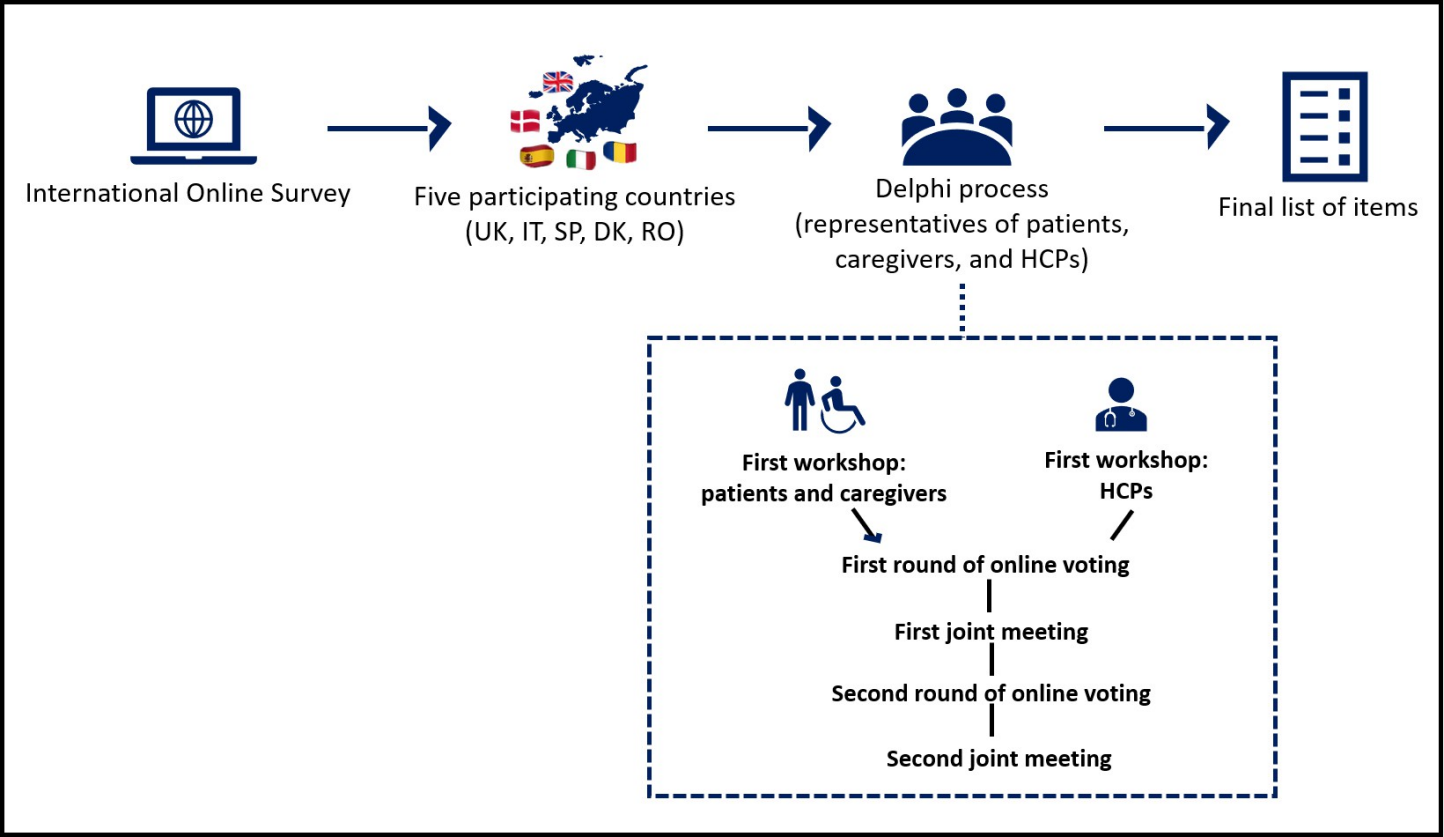
Flow-chart of the study process. UK = United Kingdom, IT = Italy, SP = Spain, DK = Denmark, RO = Romania, HCPs = Healthcare Professionals, QPIs = Quality of Performance Indicators.

The identification of the key needs, QPIs and outcomes consisted of two phases (**Fig 1**): (i) an international on-line survey open to patients, their caregivers, and HCPs distributed in five countries (UK, Italy, Spain, Denmark, Romania) participating in the AFFIRMO study [17], and (ii) a Delphi process with international patients, caregivers, and HCP representatives.

Ethical approval from local and national authorities was sought and granted in the UK (REC 21/YH/0307), Italy (015534, ref. 5308/AO/22), Spain (2021-12-15-HCUVA), and Romania (2SNI/13.01.2022). Consultation with the Danish Research Ethics committee confirmed no need for ethical approval for this type of study in Denmark.

### Phase 1: On-line survey

An online survey platform was developed by AdvicePharma to host the international survey, and the content of the patient, caregiver and HCP surveys **(S2-S4 Tables in S1 File**, respectively) were developed by a multidisciplinary project team (DAL, CT, DGL, TL and GG) composed of psychologists, geriatrician, and an AF patients’ representative. Validated questionnaires were included to assess patients quality of life (EQ-5D-3L [18]), perception of healthcare received (Healthcare Climate Questionnaire–HCCQ [19]), engagement in the healthcare process (Altarum Consumer Engagement Measure–ACE [20]; and the Patient Health Engagement Scale–PHE-s [21]), frailty (FRAIL questionnaire [22]), and medication adherence (5-item Medication Adherence Report Scale–MARS-5 [23]). In the caregiver survey, quality of life was assessed using the ED-5D-3L (ref), engagement in the healthcare process with the Caregivers Health Engagement Scale (CHE-s) [24]), and level of life changes by the Bakas Caregiving Outcome Scale (BCOS) [25]).

In the HCPs survey, data related to demographics (age, sex, racial/ethnic origin), job role and related details (e.g. medical specialty; primary, secondary or tertiary care centre), and country of residence were collected. Additionally, the survey also aimed to seek HCPs view on the importance that they attribute to patient’s engagement in the care management. The online survey was divided by groups: (i) patients, (ii) caregivers, and (iii) HCPs, each asking socio-demographic information (e.g., age, sex, level of education) and reporting specific self-reported questionnaires to identify key needs, QPIs, and key outcomes of treatment for the intended category. A list of the questionnaires used and the template of the survey can be found in Appendix 1. All three surveys were developed in English and then translated into Italian, Spanish, Danish and Romanian. Validated questionnaires that did not have a translation for the countries included in the online survey were translated by a professional company, and the back-translation was confirmed by the relevant country leads.

The survey was open to patients with AF and one or more concomitant chronic health conditions (multimorbidity), and their caregivers (dyads and non-dyads), and to HCPs managing patients with AF and one or more concomitant chronic health conditions. Exclusion criteria were as follows: (i) inability to consent, (ii) moderate or severe cognitive impairment (e.g. dementia), (iii) presence of moderate or severe conditions that may prevent completion of the survey, (iv) inability to complete the survey online, and (v) unwilling to participate in the study. Patients and caregivers were invited to participate in the survey via advertisement on a patient organisation website (Atrial Fibrillation Association, AFA), or by HCPs to patients attending clinical appointments in participating hospitals. HCPs were invited to participate by e-mail via professional networks. The first page of the online survey asked the participant to provide consent; this was mandatory prior to accessing the main survey. The online survey was distributed between 31 May 2022 and 31 January 2023 in the UK, Italy, Spain, Denmark, and Romania.

### Phase 2: Delphi process

The needs, QPIs, and outcomes identified from the survey (Phase 1) were discussed during a Delphi process [26] that involved three online meetings of approximately two hours each (via Zoom), and two rounds of independent off-line voting using the JISC survey platform. The inclusion criteria for the Delphi process were the same as the on-line survey, with the addition of being able to speak English, as a common language was required for the meetings. Participants were asked to sign and return a consent form via email. Participation required attendance at all the meetings and completion of all rounds of voting. The Delphi process ran between February and April 2023. The initial meeting was held separately for patients/caregivers and for HCPs. The patient-caregiver meeting was chaired by a patient advocate and the HCP meeting by a geriatrician, independent from the AFFIRMO project. The Chairs did not participate in the voting. A list of Delphi panel members is available in the S1 File.

After these meetings, the link to access the first round of voting was sent by email to all participants, with one week to respond. Participants were asked to score each item on a scale from 1 to 9, where 1–3 was defined as “not important at all”, 4–6 as “important but not essential”, and 7–9 as “extremely important”. Only items scored as “extremely important” (7–9 score) by the 80% or more of the participants were included in the final list. Items scored as “not important at all” (1–3 score) by 80% or more of the participants were moved to the “excluded list” (**S1 Table in S1 File**). Items where a majority decision for inclusion/exclusion (those that did not reach ≥80%) was not reached, were carried over to the next online meeting for discussion.

The second online meeting was held jointly for all participants. The results from the first round of voting were presented (included, excluded and undecided (those scored 4–6)). Following this meeting, participants were sent the link to access the second round of voting by email. The final joint meeting summarised the results of the second round of voting. Needs, QPIs and outcomes that still did not reach consensus, were scored anonymously during the meeting using VoxVote (VoxVote, The Netherlands). The final list of key needs, QPIs and outcomes was then presented to participants.

### Data analysis

Results of the online survey were reported using descriptive statistics (i.e. mean and standard deviation [SD] or median [interquartile range] for continuous variables normally or non-normally distributed, respectively; and counts/percentages for categorical data. Consensus on the inclusion/exclusion of the items presented during the Delphi process was defined in line with the GRADE guidelines [27] (details listed above).

## Results

### Online survey

A total of 1,305 participants completed the online survey. Of these, 659 (50.5%) were patients with a mean (SD) age of 70.9 (10.2) years and 348 (52.8%) female; 201 (%) were caregivers, mean (SD) age 58.3 (15.2) years and 147 (73.1%) female; and 445 (%) were HCPs, mean (SD) age 47.4 (10.6) and 257 (57.2%) female. More than half of the patients were from the UK (n = 358, 54.3%). Caregivers were mainly recruited from Spain, Romania and Italy, and HCPs were mainly recruited from the UK, Italy, Spain, and Romania. The distribution for each participant group by country is shown in **S1 Fig in S1 File**.

**Table 1** presents the characteristics of the patients who completed the online survey. Level of education among patients varied, with degree level or above being the most common (42.5%). Most patients were currently retired (78.5%), married/had a partner (68%), and lived at home with family without assistance (63.6%). When assistance was required, it was mainly informal (92.7%). Most patients reported having more than two comorbidities (45%), with 10% having more than five comorbidities. The most common comorbidities reported were other cardiovascular diseases (60.5%), hypertension (59%), and osteoarthritis (25%).

**Table 1:** Characteristics of the patients who participated in the international on-line survey.

Mean (SD), n (%)	Patient group (n = 659)
Age (years)	70.9 (10.2)
Women	348 (52.8)
Ethnicity	
White	645 (97.9)
Hispanic or Latino	6 (1.1)
Country	
UK	358 (54.3)
Italy	84 (12.7)
Spain	122 (18.5)
Romania	92 (14.0)
Denmark	3 (0.5)
Level of Education	
None	17 (2.7)
Primary	77 (12.0)
Secondary	51 (7.7)
High School*	158 (24.0)
Apprenticeship/Professional training/vocational training*	57 (8.6)
Degree level or above	280 (42.5)
Other/prefer not to say	19 (2.9)
Employment status	
Employed	102 (15.5)
Unemployed	20 (3.0)
Retired	517 (78.5)
Disability Allowance	20 (3.0)
Marital status	
Single/Never married	39 (5.9)
Married/Partnered	448 (68.0)
Widowed	111 (16.8)
Separated/Divorced	61 (9.3)
Living arrangements	
Living at home alone with no assistance	168 (25.5)
Living at home with family with no assistance	419 (63.6)
Living at home with part-time assistance	50 (7.6)
Living at home with full-time assistance	19 (2.9)
Living in long-term care facilities	3 (0.5)
If assistance is required, the caregiver is	
Formal	48 (7.3)
Informal	611 (92.7)
Smoking status	
Current	33 (5.0)
Former	281 (42.6)
Never	345 (52.4)
Comorbidities (n, %)	
Hypertension	389 (59.0)
Cardiovascular disease	399 (60.5)
Diabetes mellitus	108 (16.4)
Thyroid disease	108 (16.4)
Chronic obstructive pulmonary disease	41 (6.2)
Gastrointestinal diseases	128 (19.4)
Chronic liver disease	19 (2.9)
Kidney disease	62 (9.4)
Previous stroke	57 (8.6)
Parkinson’s disease	8 (1.2)
Multiple sclerosis	3 (0.5)
Dementia	2 (0.3)
Cognitive decline	46 (7.0)
Osteoarthritis	165 (25.0)
Osteoporosis/previous hip fracture	51 (7.7)
Rheumatoid arthritis	29 (4.4)
Chronic pain	77 (11.7)
Vision problems	119 (18.1)
Hearing problems	105 (15.9)
Cancer	40 (6.1)
Other	182 (27.6)
Number of comorbidities	
None	27 (4.0)
1–2 comorbidities	270 (41.0)
3–5 comorbidities	297 (45.0)
>5 comorbidities	66 (10.0)
Hospital visits per year, median (IQR)	0.0 (0.0–1.0)

IQR, interquartile range; SD, standard deviation

**Table 2** presents the characteristics of the caregivers who participated. Most caregivers reported spending less than 6h/day in caring activities (50.2%). Most were informal caregivers (91.5%), with more than five years as a caregiver (44.8%), mainly assisting a parent (36.8%), spouse/partner (27.9%) or other relative (25.4%). Less than half (46.8%) lived with the assisted person. The person they cared for had two or more comorbidities, predominantly cardiovascular conditions. Most assisted persons were taking more than five medications (70.6%), were able to walk independently (60.7%), but required assistance with some activities of daily living.

**Table 2:** Characteristics of the caregivers who participated in the international on-line survey.

Mean (SD), n (%)	Caregiver group (n = 201)
Age (years)	58.3 (15.2)
Women	147 (73.1)
Ethnicity	
White	199 (99.0)
Other	2 (1.0)
Country	
UK	6 (3.0)
Italy	49 (24.4)
Spain	66 (32.8)
Romania	80 (39.8)
Denmark	0 (0.0)
Level of Education	
None	2 (1.0)
Primary	23 (11.4)
Secondary	22 (11.0)
High School	40 (20.0)
Apprenticeship/Professional training/vocational training	15 (7.5)
Degree level or above	77 (35.0)
Other/prefer not to say	22 (11.0)
Time spent caregiving	
Full-time	43 (21.4)
Less than 6 h/day, daily	57 (28.4)
Less than 6h/day, not daily	101 (50.2)
Person assisted–if informal (n = 184)	
Spouse/partner	56 (27.9)
Father	31 (15.4)
Mother	43 (21.4)
Any other relative	51 (25.4)
A friend	3 (1.5)
Living with assisted person	94 (46.8)
Type of caregiver	
Formal	17(8.5)
Informal	184(91.5)
Years being caregiver	
≤1 year	50 (24.9)
2–4 years	61 (30.3)
≥5 years	90 (44.8)
Comorbidities of the assisted person	
High blood pressure	124 (61.7)
Heart disease	171 (85.1)
Diabetes	66 (32.8)
Thyroid problems	38 (18.9)
Chronic obstructive pulmonary disease	24 (11.9)
Gastrointestinal diseases	32 (15.9)
Chronic liver disease	7 (3.5)
Kidney disease	38 (18.9)
Previous stroke	31 (15.4)
Parkinson’s disease	5 (2.5)
Multiple sclerosis	1 (0.5)
Dementia	11 (5.5)
Cognitive decline	29 (14.4)
Osteoarthritis	41 (20.4)
Osteoporosis/previous hip fracture	22 (10.9)
Rheumatoid arthritis	14 (7.0)
Chronic pain	22 (10.9)
Vision problems	41 (20.4)
Hearing problems	42 (20.9)
Cancer	22 (10.9)
Other	27 (13.4)
Number of comorbidities of the assisted person	
None	5 (2.5)
1–2 comorbidities	79 (39.3)
3–5 comorbidities	87 (43.3)
>5 comorbidities	30 (15.0)
Number of medications taken by the assisted person	
None	5 (2.5)
1 to 2	10 (5.0)
3 to 4	44 (21.9)
5 or more	142 (70.6)
Mobility level of the assisted person	
Can walk independently	122 (60.7)
Walk with a cane/walking stick	37 (18.4)
Walk with a walker/Zimmer-frame	28 (13.9)
Moves around with a wheelchair	5 (2.5)
Confined at home, mostly lying on the bed	9 (4.5)
Activities that require assistance	
Eating	37 (18.4)
Bathing	81 (40.3)
Dressing	45 (22.4)
Toileting	35 (17.4)
Transferring	141 (70.1)

SD, standard deviation

**Table 3** presents the characteristics of the HCPs that completed the online survey. Most HCP respondents were medical doctors (73.5%), either cardiologists (45.2%) or geriatricians (31.7%). Years of practice varied from less than five years (23.6%) to more than 30 years (20.9%). Most worked in secondary (36%) or tertiary care (38.7%) and managed two to five patients with AF per week, and the most common comorbidities were cardiovascular diseases (92.6%).

**Table 3:** Characteristics of the healthcare professionals who participated in the international on-line survey.

Mean (SD), n (%)	Healthcare professional group (n = 445)
Age (years)	47.4 (10.6)
Women	257 (57.8)
Country	
UK	72 (16.2)
Italy	127 (28.5)
Spain	120 (27.0)
Romania	100 (22.5)
Denmark	26 (5.8)
Occupation	
Medical doctor	327 (73.5)
Nurse	110 (24.7)
Other	8 (1.8)
Specialty	
Cardiology	201 (45.2)
GP	17 (3.8)
Geriatrics/Elderly care	141 (31.7)
Haematology	8 (1.8)
Internal medicine	38 (8.5)
Other	40 (9.0)
Years of practice	
0 to 5	105 (23.6)
6 to 10	65 (14.6)
11 to 20	105 (23.6)
21 to 30	77 (17.3)
>30	93 (20.9)
Care sector	
Primary care	113 (25.4)
Secondary care	160 (36.0)
Tertiary care	172 (38.7)
University Hospital	
Yes	337 (75.7)
No	108 (24.3)
Regularly working with patients with chronic condition(s)	
Yes	430 (96.6)
Sometimes	13 (2.9)
No	2 (0.4)
Patients with AF managed per week	
0 to 1	45(10.1)
2 to 5	231 (51.9)
6 to 10	81 (18.2)
>10	88 (19.8)
Most frequently managed conditions	
Cardiovascular	412 (92.6)
Diabetes	172 (38.7)
Endocrinologic diseases	6 (1.3)
Respiratory diseases	136 (30.6)
Chronic liver diseases	7 (1.6)
Gastrointestinal diseases	13 (2.9)
Kidney diseases	73 (16.4)
Cerebrovascular diseases	71 (16.0)
Neurologic diseases	19 (4.3)
Minor/major cognitive disorders	86 (19.3)
Osteoarticular diseases	25 (5.6)
Rheumatologic diseases	9 (2.0)
Chronic pain	16 (3.6)
Vision problems	0 (0.0)
Hearing problems	0 (0.0)
Other	12 (2.7)
Most represented age group	
<60 years	11 (2.5)
60–70 years	108 (24.3)
71–80 years	201 (45.2)
>80 years	125 (28.1)
Assisted patients with AF and at least one other chronic condition	
0 to 10%	7 (1.6)
11 to 30%	28 (6.3)
31 to 50%	56 (12.6)
51 to 80%	141 (31.7)
>80%	213 (47.9)

AF, atrial fibrillation; SD, standard deviation

The list of needs, QPIs and key outcomes identified by the online survey (n = 53) are shown in **S1 Table in S1 File**.

### Delphi process

A total of 21 participants joined the Delphi panel: 8 patients, 2 caregivers and 11 HCPs. More than half of the participants were men (n = 11, 52%). Due to the requirement of being able to speak English, all patients and caregivers were from the UK (n = 10, 48%). Among the HCPs, there were three participants each from Italy and Denmark, two each from Spain and Romania, and one from Australia. Of this group, three were nurses and eight were physicians, with four specialising in geriatrics and/or internal medicine and four in cardiology.

**Fig 2:**
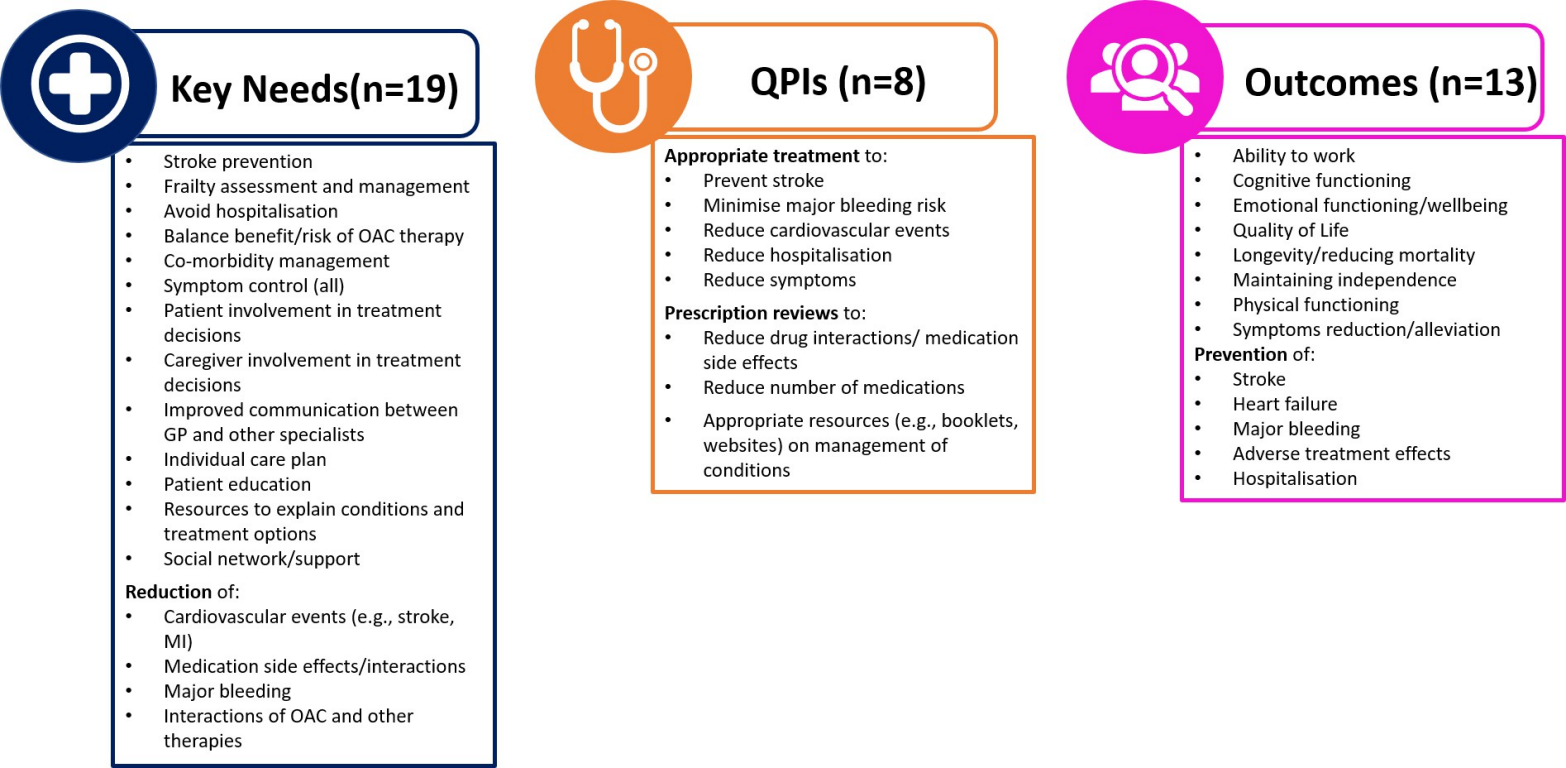
Summary of the items that reached consensus in during the Delphi process. OAC = oral anti-coagulant, QPIs = quality of performance indicators. Note: some rewording has been made in the Figure compared to the list in Table 4 for presentation purposes.

The key needs, QPIs and key outcomes identified by the online survey (**S1 Table in S1 File**) were presented to the Delphi panel at the initial meetings. Forty items reached ‘consensus in’ during the process and were included in the final list. These were grouped into 19 key needs, eight QPIs and 13 key outcomes (**Table 4 and Fig 2**).

**Table 4:** Final list of key needs, quality performance indicators and key outcomes reaching consensus.

Key Needs (n = 19)	Quality Performance Indicators (n = 8)	Key outcomes (n = 13)
• Stroke prevention • Assessment and management of frailty • Avoid hospitalization • Balance benefit/risk ratio due to anticoagulant treatment • Caregiver involvement in treatment decisions • Co-morbidity management • Control AF symptoms • Control heart failure symptoms • Control possible interactions between anticoagulant and other ongoing treatments* • Improved communication between GP and other specialists • Individual care plan • Materials to explain conditions and treatment options • Patient education • Patient involvement in treatment decisions • Reducing cardiovascular events (e.g., stroke, heart attack) • Reducing medication side effects • Reduction of major bleeding • Social network/support • Symptoms control (all symptoms)	• Appropriate prescription review to reduce drug interaction and medication side effects • Appropriate prescription review to reduce the number of medications • Appropriate resources (e.g., booklets, websites) to provide information on the management of the conditions and on medical recommendation to patients and their caregiver • Appropriate stroke prevention/treatment • Appropriate treatment to reduce the occurrence of cardiovascular events • Appropriate treatment to reduce the risk of major bleeding • Appropriate treatment to reduce/avoid hospitalization • Reduction/alleviation of symptoms	• Ability to work • Cognitive functioning • Emotional functioning/wellbeing • Longevity/reducing mortality • Maintaining independence • Physical functioning • Preventing heart failure • Preventing major bleeding • Preventing/reducing adverse treatment effects • Preventing/reducing hospitalization • Quality of Life • Stroke prevention • Symptoms reduction/alleviation

*described in Fig 2 as “Interaction of OAC (oral anti-coagulant) and other therapies”.

## Discussion

In our study, we aimed to identify the key needs, QPIs, and outcomes relevant to multimorbid patients with AF, their caregivers, and the HCPs that manage their health, collecting data from an international online survey and reaching consensus through a Delphi panel with international stakeholders.

The online survey identified a preliminary list of 53 items, which were reduced to 40 items during the Delphi process and were included in the final list of key needs, quality performance indicators and outcomes. Of these, 19 key needs were identified including stroke prevention, symptom control, reducing adverse events/medication side effects, and improving patient education and HCP-patient communication. Eight QPIs were identified including appropriate treatment and medications relating to stroke prevention and to reduce adverse events (bleeding and cardiovascular events) and medication reviews to ensure optimal management and to reduce medication side effects, and provision of appropriate education tools for patients and caregivers. Thirteen key outcomes were identified including the ability to work, quality of life, preventing hospitalisation, stroke, and cardiovascular diseases, reducing adverse events/medication side effects, and symptom management.

The importance of a clear definition of key needs and outcomes in clinical research and practice has been previously highlighted [26, 28], and international initiatives such as the “Core Outcomes Measures in Effectiveness Trials” (COMET) Initiative [29] have established guidelines on how to construct core outcomes sets and how to involve key stakeholders (patients, caregivers and HCPs) in the process. However, until now there has been no comprehensive list of key needs, QPIs and outcomes for patients with AF and other concomitant long-term health conditions. The identification of a set of key items that are relevant for the management of patients with AF and multimorbidity from the AFFIRMO study will help to inform and redirect the healthcare management of these patients toward a more holistic and unified approach [30], integrating the needs of patients and of the people that care for them into consideration, and focusing on QPIs and outcomes of importance to them. This list of items has informed the outcomes of the AFFIRMO clinical trial, which will hopefully help to improve the clinical management of patients with AF in the context of multimorbidity.

### Strengths and limitations

Our study collected data from a large international cohort of 1,305 patients, caregivers and HCPs from five European countries, identifying key needs, QPIs and outcomes of healthcare that are important to a vast range of stakeholders, also representing all regions of Europe (North, South and East Europe). The cohort was representative of the typical AF population in terms of age and comorbidities and included 57% females. The use of a consolidated methodology, such as the Delphi process, to reach consensus on the core items to report in the final list is a strength of our study. Involvement of patients’ and caregiver representatives in the Delphi process, and also in the inclusion of a patient advocate as co-chair of the joint sessions, has mitigated the potential “medical-centred” discussion that could have arisen from a group of HCPs only. Patients and caregivers have been able to express their views, sometimes with constructive criticism on the medicalised approach they have experienced, thus improving the quality of the discussion during the Delphi process.

Some limitations of the study are noteworthy. Patients and caregivers from Denmark and caregivers from the UK were under-represented in the online survey, mainly due to difficulties in engaging these groups in the respective countries. However, overall 50% and 15% of the participants were patients and caregivers, respectively, and therefore, we believe the study has captured their ‘voice’ and there is no reason to believe that the opinions and insights of patients in Denmark and caregivers in the UK and Denmark would differ significantly from those captured in this study. The presence of only UK patient and caregiver participants in the Delphi process (due to the need of a common language for the discussion) is a limitation, which may have presented viewpoints related to one specific healthcare system, and may have influenced the key needs, QPIs, and outcomes selected. However, HCPs from five countries contributed to the Delphi process which ensured diversity in healthcare system perspectives. Further, the Delphi workshops discussed the needs, QPIs and outcomes identified by patients, caregivers and HCPs from five European healthcare systems via the on-line surveys, which minimises the possible UK-biased perspective of healthcare. Moreover, patients and caregivers who participated in the survey were mainly of White ethnicity which may reduce the generalisability of our findings.

## Conclusions

Forty items, divided into 19 key needs, eight QPIs, and 13 key outcomes, relevant to patients with AF and multimorbidity, their caregivers and HCPs were identified from an international survey and selected through a Delphi process. This list has informed the outcomes of the AFFIRMO clinical trial, which aims to direct the care of these patients to a more patient-centred approach.

## Supporting information

S1 File Supplementary materials. List of AFFIRMO study investigators. List of Delphi Panel members. S1 Table. Full list of items identified from the survey that were scored in the Delphi process. S2 Table. Content of the online survey for patients. S3 Table. Content of the online survey for caregivers. S4 Table. Content of the online survey for healthcare professionals.
(DOCX)

## List of abbreviations

ABC: Atrial Fibrillation Better Care
ACE: Altarum Consumer Engagement Measure
AF: Atrial Fibrillation
AFFIRMO Study: Atrial Fibrillation Integrated Approach in Frail, Multimorbid, and Polymedicated Older People Study
BCOS: Bakas Caregiving Outcome Scale
CHE-s: Caregivers Health Engagement Scale
COMET Initiative: Core Outcomes Measures in Effectiveness Trials Initiative
GRADE guidelines: Grading of Recommendations, Assessment, Development, and Evaluations guidelines
HCCQ: Healthcare Climate Questionnaire
HCPs: Healthcare Professionals
MARS-5: 5-item Medication Adherence Report Scale
PHE-s: Patient Health Engagement
QPIs: Quality of Performance Indicators

## References

[1] Ceornodolea AD, Bal R, Severens JL. Epidemiology and Management of Atrial Fibrillation and Stroke: Review of Data from Four European Countries. Stroke Research and Treatment. 2017;2017:8593207. doi: 10.1155/2017/8593207 28634569 PMC5467327

[2] Lip GY. The ABC pathway: an integrated approach to improve AF management. Nature Reviews Cardiology. 2017;14(11):627–8. doi: 10.1038/nrcardio.2017.153 28960189

[3] Chao TF, Joung B, Takahashi Y, Lim TW, Choi EK, Chan YH, et al. 2021 Focused Update Consensus Guidelines of the Asia Pacific Heart Rhythm Society on Stroke Prevention in Atrial Fibrillation: Executive Summary. Thromb Haemost. 2022;122(1):20–47. doi: 10.1055/s-0041-1739411 34773920 PMC8763451

[4] Proietti M, Lip GY, Laroche C, Fauchier L, Marin F, Nabauer M, et al. Relation of outcomes to ABC (Atrial Fibrillation Better Care) pathway adherent care in European patients with atrial fibrillation: an analysis from the ESC-EHRA EORP Atrial Fibrillation General Long-Term (AFGen LT) Registry. Europace. 2020;23(2):174–83. 10.1093/europace/euaa274 33006613

[5] Hindricks G, Potpara T, Dagres N, Arbelo E, Bax JJ, Blomstro C, et al. 2020 ESC Guidelines for the diagnosis and management of atrial fibrillation developed in collaboration with the European Association of Cardio-Thoracic Surgery (EACTS). European heart journal. 2020;42(5):373–498. 10.1093/eurheartj/ehaa612 32860505

[6] Xu X, Mishra GD, Jones M. Evidence on multimorbidity from definition to intervention: An overview of systematic reviews. Ageing Research Reviews. 2017;37:53–68. doi: 10.1016/j.arr.2017.05.003 28511964

[7] Barnes PJ. Mechanisms of development of multimorbidity in the elderly. European Respiratory Journal. 2015;45(3):790–806. doi: 10.1183/09031936.00229714 25614163

[8] Vanbeselaere V, Truyers C, Elli S, Buntinx F, De Witte H, Degryse J, et al. Association between atrial fibrillation, anticoagulation, risk of cerebrovascular events and multimorbidity in general practice: a registry-based study. BMC Cardiovasc Disord. 2016;16:1–12. 27021333 10.1186/s12872-016-0235-1 PMC4810573

[9] Chamberlain AM, Alonso A, Gersh BJ, Manemann SM, Killian JM, Weston SA, et al. Multimorbidity and the risk of hospitalization and death in atrial fibrillation: A population-based study. Am Heart J. 2017;185:74–84. doi: 10.1016/j.ahj.2016.11.008 28267478 PMC5343767

[10] Rijken M, Hujala A, van Ginneken E, Melchiorre MG, Groenewegen P, Schellevis F. Managing multimorbidity: Profiles of integrated care approaches targeting people with multiple chronic conditions in Europe. Health Policy. 2018;122(1):44–52. doi: 10.1016/j.healthpol.2017.10.002 29102089

[11] Lip GYH. Managing high-risk atrial fibrillation patients with multiple comorbidities. International Journal of Arrhythmia. 2023;24(1):4.

[12] Proietti M, Esteve-Pastor MA, Rivera-Caravaca JM, Roldán V, Roldán Rabadán I, Muñiz J, et al. Relationship between multimorbidity and outcomes in atrial fibrillation. Experimental Gerontology. 2021;153:111482. doi: 10.1016/j.exger.2021.111482 34303775

[13] Woo BFY, Bulto LN, Hendriks JML, Lim TW, Tam WWS. The information needs of patients with atrial fibrillation: A scoping review. J Clin Nurs. 2023;32(9–10):1521–33. doi: 10.1111/jocn.15993 34390046

[14] Arbelo E, Aktaa S, Bollmann A, D’Avila A, Drossart I, Dwight J, et al. Quality indicators for the care and outcomes of adults with atrial fibrillation. Europace. 2021;23(4):494–5. doi: 10.1093/europace/euaa253 32860039

[15] Stewart M. Towards a global definition of patient centred care: the patient should be the judge of patient centred care. 2001;322(7284):444–5. 10.1136/bmj.322.7284.444 PMC1119673 11222407

[16] Bayliss EA, Bonds DE, Boyd CM, Davis MM, Finke B, Fox MH, et al. Understanding the Context of Health for Persons With Multiple Chronic Conditions: Moving From What Is the Matter to What Matters. The Annals of Family Medicine. 2014;12(3):260–9. doi: 10.1370/afm.1643 24821898 PMC4018375

[17] Johnsen SP, Proietti M, Maggioni AP, Lip GYH. A multinational European network to implement integrated care in elderly multimorbid atrial fibrillation patients: the AFFIRMO Consortium. Eur Heart J. 2022;43(31):2916–8. doi: 10.1093/eurheartj/ehac265 35598035

[18] EuroQol G. EuroQol—a new facility for the measurement of health-related quality of life. Health Policy. 1990;16(3):199–208. doi: 10.1016/0168-8510(90)90421-9 10109801

[19] Williams GC, Grow VM, Freedman ZR, Ryan RM, Deci EL. Motivational predictors of weight loss and weight-loss maintenance. Journal of personality and social psychology. 1996;70(1):115. doi: 10.1037//0022-3514.70.1.115 8558405

[20] Duke CC, Lynch WD, Smith B, Winstanley J. Validity of a New Patient Engagement Measure: The Altarum Consumer Engagement (ACE) Measure. Patient. 2015;8(6):559–68. doi: 10.1007/s40271-015-0131-2 26097010 PMC4662956

[21] Graffigna G, Barello S, Bonanomi A, Lozza E. Measuring patient engagement: development and psychometric properties of the Patient Health Engagement (PHE) Scale. Front Psychol. 2015;6:274. doi: 10.3389/fpsyg.2015.00274 25870566 PMC4376060

[22] Gleason LJ, Benton EA, Alvarez-Nebreda ML, Weaver MJ, Harris MB, Javedan H. FRAIL Questionnaire Screening Tool and Short-Term Outcomes in Geriatric Fracture Patients. J Am Med Dir Assoc. 2017;18(12):1082–6. doi: 10.1016/j.jamda.2017.07.005 28866353 PMC6611671

[23] Horne R, Weinman J. Self-regulation and self-management in asthma: exploring the role of illness perceptions and treatment beliefs in explaining non-adherence to preventer medication. Psychology and Health. 2002;17(1):17–32.

[24] Barello S, Castiglioni C, Bonanomi A, Graffigna G. The Caregiving Health Engagement Scale (CHE-s): development and initial validation of a new questionnaire for measuring family caregiver engagement in healthcare. BMC Public Health. 2019;19(1):1562. doi: 10.1186/s12889-019-7743-8 31771546 PMC6880352

[25] Bakas T, Champion V. Development and psychometric testing of the Bakas Caregiving Outcomes Scale. Nursing Research. 1999;48(5):250–9. doi: 10.1097/00006199-199909000-00005 10494909

[26] Biggane AM, Brading L, Ravaud P, Young B, Williamson PR. Survey indicated that core outcome set development is increasingly including patients, being conducted internationally and using Delphi surveys. Trials. 2018;19(1):1–6. 29454368 10.1186/s13063-018-2493-y PMC5816387

[27] Schünemann HJ, Oxman AD, Brozek J, Glasziou P, Bossuyt P, Chang S, et al. GRADE: assessing the quality of evidence for diagnostic recommendations. Evid Based Med. 2008;13(6):162–3. doi: 10.1136/ebm.13.6.162-a 19043023

[28] Boers M, Kirwan JR, Wells G, Beaton D, Gossec L, d’Agostino M-A, et al. Developing core outcome measurement sets for clinical trials: OMERACT filter 2.0. Journal of clinical epidemiology. 2014;67(7):745–53. doi: 10.1016/j.jclinepi.2013.11.013 24582946

[29] Prinsen CA, Vohra S, Rose MR, King-Jones S, Ishaque S, Bhaloo Z, et al. Core Outcome Measures in Effectiveness Trials (COMET) initiative: protocol for an international Delphi study to achieve consensus on how to select outcome measurement instruments for outcomes included in a ‘core outcome set’. Trials. 2014;15(1):1–7. 24962012 10.1186/1745-6215-15-247 PMC4082295

[30] Romiti GF, Proietti M, Vitolo M, Bonini N, Fawzy AM, Ding WY, et al. Clinical complexity and impact of the ABC (Atrial fibrillation Better Care) pathway in patients with atrial fibrillation: a report from the ESC-EHRA EURObservational Research Programme in AF General Long-Term Registry. BMC medicine. 2022;20(1):326. doi: 10.1186/s12916-022-02526-7 36056426 PMC9440492

