# Supplementary material for: Key needs, quality performance indicators and outcomes for patients with atrial fibrillation and multimorbidity: The AFFIRMO study
Source: PLoS One. 2024 Sep 10;19(9):e0310106. doi: 10.1371/journal.pone.0310106 (PMC12139655; doi:10.1371/journal.pone.0310106)
Supplement: S1 File — List of AFFIRMO study investigators. List of Delphi Panel members. S1 Table. Full list of items identified from the survey that were scored in the Delphi process. S2 Table. Content of the online survey for patients. S3 Table. Content of the online survey for caregivers. S4 Table. Content of the online survey for healthcare professionals. (DOCX) [file pone.0310106.s001.docx]

**Supplementary Material**

**List of AFFIRMO Study Investigators**

Søren Påske Johnsen, Gregory Lip, Martin O’Flaherty, Mirko Petrovic, Davide Liborio Vetrano, Laura Vivani, Guendalina Graffigna, Aldo Pietro Maggioni, Dipak Kalra, John Ainsworth, Peter Brønnum Nielsen, Berit Hviderg Christensen, Gitte Berg Jensen, Riccardo Proietti, Carrol Gamble, Iain Buchan, Christodoulos Kypridemos, Brendan Collins, Mirko Petrovic, Delphine De Smedt, Stefanie De Buyser, Cheima Amrouch, Amaia Calderón-Larrañaga, Lu Dai, Stefania Maggi, Marianna Noale, Gheorghe-Andrei Dan, Anca Rodica Dan, Elisabeta Badila, Raluca Alexandra Popescu, Adrian Buzea, Nicola Ferri, Alessandra Buja, Giuseppe Sergi, Vincenzo Stefano Rebba, Tatjana Potpara, Miroslav Mihajlovic, Veronica Christofidis, Silvia Ananstasia, Dimitriya Bozukova, Alessandro Ferri, Gehad Shehata, Nadia Rosso, Marco Cicerone, Giuseppe Grandolfo, Jacek Marczyk, Georg Ruppe, Graziano Onder, Federica Censi, Robero Da Cas, Cecilia Damiano, Caterina Bosio, Lorenzo Palamenghi, Serena Barello, Andrea Lorimer, Donata Lucci, Marco Proietti, Nathan Lea, Charlotte Stockton-Powdrell, Alam Sanaullah, Francisco Marín Ortuño, José Miguel Rivera-Caravaca, Pablo Gil Pérez, Mariya Tokmakova, Angel Dzhambov

**LIST OF DELPHI PANEL MEMBERS**

Asangaedem Akpan (Chair), Susan G. Aubrey, Peter Krogh Brynningsen, Ioana Cristina Daha, Paul Everington, Gillian Feast, Stefano Fumagalli, Tracy Goodman (Co-Chair), Andreea Ivanescu, John Knight, Trudie C.A Lobban (Co-Chair), Ivan Lowe, Daisy Lussier, Jeroen Mendruis, Rosemary North, Marco Proietti, Valeria Raparelli, Signe Risom, José Miguel Rivera Caravaca, Danny Roberts, Vanessa Roldán, Paul Vickery, Anna Maria Wardle

| **Supplementary Table 1.** Full list of items identified from the survey that were scored in the Delphi process. | | |
| --- | --- | --- |
| **Key Needs (n=27)** | **Quality Performance Indicators (n=9)** | **Key outcomes (n=17)** |
| - Assessment of frailty* - Avoid hospitalization - Balance benefit/risk ratio due to anticoagulant treatment - Caregiver involvement in treatment decisions - Co-morbidity management - Control AF symptoms - control heart failure symptoms - Control possible interactions between anticoagulant and other ongoing treatments - Improved communication between GP and other specialists - Individual care plan - Management of frailty* - Managing the impact on dementia - Materials to explain conditions and treatment options - Patient education - Patient involvement in treatment decisions - Reduce number of medications - Reducing anxiety - Reducing cardiovascular events (e.g., stroke, heart attack) - Reducing medication side effects - Reducing number of medical appointments - Reduction of major bleeding - Social network/support - Social/leisure activities - Stroke prevention - Sufficient information on management plan - Support for caregivers - Symptoms control (all symptoms) | - Appropriate prescription review to reduce drug interaction and medication side effects - Appropriate prescription review to reduce the number of medications - Appropriate resources (e.g., booklets, websites) to provide information on the management of the conditions and on medical recommendation to patients and their caregivers - Appropriate strategy in place to reduce the number of medical appointments - Appropriate stroke prevention/treatment - Appropriate treatment to reduce the occurrence of cardiovascular events - Appropriate treatment to reduce the risk of major bleeding - Appropriate treatment to reduce/avoid hospitalization - Reduction/alleviation of symptoms | - Ability to work - Cognitive functioning - Emotional functioning/wellbeing - Exercise tolerance - Longevity/reducing mortality - Maintaining independence - Pain control/relief - Physical functioning - Preventing heart failure - Preventing major bleeding - Preventing/reducing adverse treatment effects - Preventing/reducing hospitalization - Quality of Life - Reducing medication side effects - Social/leisure functioning - Stroke prevention - Symptoms reduction/alleviation |

*these items were merged in the single item “Assessment and management of frailty” for the final list.

Items in red were excluded from the final list.

## Supplementary Table 2. Content of the online survey for patients.

| *Introduction*  *We are asking the following questions to find out about your health conditions, how they affect your daily activities and quality of life. At the end of the questionnaire, there are a few questions about your age, sex, and marital status etc. This information will help us to describe the people who have taken part in this survey to show that it represents a range of people who suffer from long-term health conditions.*  Do you have any of the following conditions? Tick **ALL** that apply:   - High blood pressure - Heart disease - Diabetes - Thyroid problems - Chronic obstructive pulmonary disease (COPD) - Gastrointestinal diseases *(e.g.* *gastritis, gastroesophageal reflux disease, diverticular disease*) - Chronic liver disease - Kidney disease - Previous stroke - Parkinson’s disease - Multiple sclerosis - Dementia - Cognitive decline (*e.g. memory problems not impacting daily activities*) - Osteoarthritis - Osteoporosis/previous hip fracture - Rheumatoid arthritis - Chronic pain - Vision problems - Hearing problems - Cancer - Other: __________________________   Which condition affects your health the most from the list above?_________________________  How many times have you been hospitalised in the last year? ____  Overall, what outcomes from the healthcare you receive are most important to you (max. 3 answers allowed)?  improvement of my quality of life  maintenance of my independence in daily life  live longer  pain reduction/relief  have less need for health care  maintenance of social and leisure activities  improvement of mental/emotional health  be able to work  other (please specify): _______________________________  What are the main problems with managing your health (max. 3 answers allowed)?  high number of medical appointments  difficulties in contacting/seeing a medical doctor  too many medications to take  having lots of health problems  not having enough financial resources  travel to medical appointments  mobility problems  anxiety/worry about my health  not having anyone to help  not understanding the medical recommendations clearly  having problems getting in contact with the doctor/healthcare team  not having the opportunity to talk freely with the doctor about doubts and fears about the health conditions and treatment  other (please specify): _______________________________ |
| --- |
| ***next page*** |
| ****Here the list of questionnaires included in the online survey following this order.**  *EQ-5D-3L(1)*  ***next page***  *FRAIL questionnaire(2)* |
| ***next page***  *PHE-s(3)*  ***next page***  *HCCQ – short version(4)* |
| ***next page***  (*Set of Brief Screening Questions*)  *When answering the questions below, please select the answers that best represent your response.* |
| How often do you have problems learning about your medical condition because of difficulty understanding written information?   \| Never \| Occasionally \| Sometimes \| Often \| Always \| \| --- \| --- \| --- \| --- \| --- \|   How often do you have someone help you read hospital materials?   \| Never \| Occasionally \| Sometimes \| Often \| Always \| \| --- \| --- \| --- \| --- \| --- \|   How confident are you filling out medical forms by yourself?   \| Not confident at all \| Not so confident \| Neutral \| Somewhat confident \| Very confident \| \| --- \| --- \| --- \| --- \| --- \| |
|  |
| *MARS-5(5)*  ***next page***  *ACE Measure(6)* |
| ***next page***  Age ____ years  Sex   - Female - Male   Living arrangements   - Living at home alone with no assistance - Living at home with family with no assistance - Living at home with part-time assistance - Living at home with full-time assistance - Living in long-term care facilities   If assistance is needed, is the caregiver   - Informal (e.g. family member; not paid) - Formal (paid)   Marital status   - Single/never married - Married/partnered - Widowed - Separated/divorced   Ethnicity   - White British - White Irish - Gypsy or Irish Traveller - Any other White background - White and Black Caribbean - White and Black African - White and Asian - Any other mixed/multiple ethnic background - Indian - Pakistani - Bangladeshi - Chinese - Any other Asian background - Black African - Black Caribbean - Any other Black background - Arab - Other (please specify)   What is the highest degree or level of education you have completed?   - Degree level or above (Bachelor’s degree, Master’s degree, NVQ level 4, Professional Qualifications, etc.) - AS, A level or equivalent - GCSEs, O levels or Equivalent, - NVQ or equivalent - Apprenticeship - No qualifications (no academic or professional qualifications) - Other (please specify)   Current employment status:   - Employed - Unemployed - Retired - Disability allowance   Smoking habit   - Current smoker - Former smoker (for at least one year) - Never smoker |

**Due to copyright, the questionnaires cannot be reproduced. The reference to each questionnaire is provided.

## Supplementary Table 3. Content of the online survey for caregivers.

| *We are asking the following questions to find out about the person you provide care for, and how being a caregiver affects you. At the end of the questionnaire, there are a few questions about your age, sex, ethnicity, and caregiving arrangements. This information will help us to describe the people who provide care.* |
| --- |
| Which of the following health conditions does the person you care for have? Tick ALL that apply:   - High blood pressure - Heart disease - Diabetes - Thyroid problems - Chronic obstructive pulmonary disease/COPD - Gastrointestinal diseases *(e.g.* *gastritis, gastroesophageal reflux disease, diverticular disease*) - Chronic liver disease - Kidney disease - Previous stroke - Parkinson’s disease - Multiple sclerosis - Dementia - Cognitive decline (*e.g. memory problems not impacting daily activities*) - Osteoarthritis - Osteoporosis/previous hip fracture - Rheumatoid arthritis - Chronic pain - Vision problems - Hearing problems - Cancer - Other: __________________________   In your view, which condition affects the health of the person you assist the most from the list above?  Which health condition of the person you assist causes you the most difficulty in providing care?  How many different medications does the assisted person take?   - 0 - 1-2 - 3-4 - 5 or more   How many times was the assisted person hospitalised in the past year? ____  Which is the mobility level of the person you provide care for?   - Can walk independently - Walks with a cane/walking stick - Walks with a walker/Zimmer-frame - Moves around with a wheelchair - Confined at home, mostly lying on the bed   Does the person you provide care for need help in any of the following activities?   - Eating - Bathing - Dressing - Toileting - Transferring   In your opinion, what health outcomes are the most important for the person you provide care for (max. 3 answers allowed)?  improvement of quality of life  maintenance of independence in daily life  live longer  pain reduction/relief  have less need for health care  maintenance of social and leisure activities  improvement of mental/emotional health  be able to work  other (please specify): _______________________________  What are the main problems with managing the health of the person you provide care for (max. 3 answers allowed)?  high number of medical appointments  difficulties in contacting/seeing a medical doctor  too many medications to manage  too many health problems to manage  not having enough financial resources  travel to medical appointments  anxiety/worry of my health  anxiety/worry for the health of the assisted person  not having anyone else to help  the responsibility of caring for someone else  mobility problems  do not understand medical recommendations clearly  having my own health problems  Having Problems getting in contact with the doctor/healthcare team  Not having the opportunity to talk freely with the doctor about doubts and fears about the health conditions and treatment  other (specify): _______________________________  ***next page***  ****Here the list of questionnaires included in the online survey following this order.**  *Bakas Caregiving Outcomes Scale(7)* |
| ***next page***  *CHE-s(8)* |
| ***next page***  *EQ-5D-3L(1)* |
| ***next page***  (*Further Questions*)  *In the questions below, please select the answers that better represent your response.*  How often do you have problems learning about your medical conditions, or the medical conditions of the person you care for because of difficulty understanding written information?   \| Never \| Occasionally \| Sometimes \| Often \| Always \| \| --- \| --- \| --- \| --- \| --- \|   How often do you have someone help you read hospital materials?   \| Never \| Occasionally \| Sometimes \| Often \| Always \| \| --- \| --- \| --- \| --- \| --- \|   How confident are you filling out medical forms by yourself?   \| Never \| Occasionally \| Sometimes \| Often \| Always \| \| --- \| --- \| --- \| --- \| --- \|   Overall, how would you rate the healthcare services received for the management of the long-term health conditions of the assisted person? Please rate this on the scale below 0 (poor) to 10 (excellent) by circling a number   \| **0** \| **1** \| **2** \| **3** \| **4** \| **5** \| **6** \| **7** \| **8** \| **9** \| **10** \| \| --- \| --- \| --- \| --- \| --- \| --- \| --- \| --- \| --- \| --- \| --- \|   Please explain why you gave this score? _______________________________  Who is the main healthcare provider that coordinates care for the person you assist?   - General Practitioner/Family doctor - Geriatrician - District/community nurse - Other (please specify) ____________   ***next page***  Age ____ years  Sex   - Female - Male   Which type of caregiver are you?   - Informal (e.g. family member; not paid) - Formal (paid)   If you are an informal caregiver, please specify if the assisted person is:   - your spouse/partner - your father - your mother - a relative other than your mother or father - a friend   Do you live with the person you provide care for:   - Yes - No   How much time do you spend in caregiving:   - I am a full-time caregiver (I have no other occupation than caregiving) - Less than 6 h/day, every day of the week - Less than 6 h/day, NOT every day of the week   How many years have you been a caregiver:   - 1 year or less - 2-4 years - ≥5 years   Ethnicity   - White British - White Irish - Gypsy or Irish Traveller - Any other White background - White and Black Caribbean - White and Black African - White and Asian - Any other mixed/multiple ethnic background - Indian - Pakistani - Bangladeshi - Chinese - Any other Asian background - Black African - Black Caribbean - Any other Black background - Arab - Other (please specify)   What is the highest degree or level of education you have completed?   - Degree level or above (Bachelor’s degree, Master’s degree, NVQ level 4, Professional Qualifications, etc.) - AS, A level or equivalent - GCSEs, O levels or Equivalent, - NVQ or equivalent - Apprenticeship - No qualifications (no academic or professional qualifications) - Other (please specify) |

***Due to copyright, the questionnaires cannot be reproduced. The reference to each questionnaire is provided.*

## Supplementary Table 4. Content of the online survey for healthcare professionals.

| Age ____ years  Sex   - Female - Male   Ethnicity   - White British - White Irish - Gypsy or Irish Traveller - Any other White background - White and Black Caribbean - White and Black African - White and Asian - Any other mixed/multiple ethnic background - Indian - Pakistani - Bangladeshi - Chinese - Any other Asian background - Black African - Black Caribbean - Any other Black background - Arab - Other (please specify)   Country   - UK - Spain - Denmark - Italy - Romania   What is your occupation?   - Medical Doctor - Nurse - Pharmacist - Occupational Therapist - Other (please specify)____________________ |
| --- |
| Which of the following best describes your working position? (only for MD)   - Self-employed - General Practitioner - Medical resident - Consultant - Hospital doctor working in inpatient clinic - Hospital doctor working in outpatient clinic - Chief of department - Other ____________________________   Specialty:   - Cardiology - Internal medicine - Geriatrics/Elderly Care - Haematology - General Practitioner - Other (please specify) __________   How many years have you been in practice since completing your degree?   - 0-5 years - 6-10 years - 11-20 years - 21-30 years - >30 years   In which setting, are you currently providing care:   - Primary care centre - Secondary care centre - Tertiary care centre   If working in a hospital, is it a University hospital:   - Yes - No |
| Do you work with patients with chronic conditions regularly (1-2 times per week)?   - Yes - No - Sometimes   Which are the chronic conditions that you manage most frequently? Please, select all that apply (up to 3):   - Cardiovascular diseases - Diabetes - Endocrinologic diseases (other than diabetes) - Respiratory diseases - Chronic liver diseases - Gastrointestinal diseases - Kidney diseases - Cerebrovascular diseases - Neurologic diseases (other than cognitive disorders) - Minor/major cognitive disorders - Osteoarticular diseases - Rheumatologic diseases - Chronic pain - Vision problems - Hearing problems - Cancer - Other (please specify): __________________________   On average, how many patients with atrial fibrillation do you manage per week?   - 0-1 - 2-5 - 6-10 - >10   On average, which age group is most represented by the patients with atrial fibrillation that you usually manage?   - <60 years - 60-70 years - 71-80 years - >80 years   On average, in your daily practice, what proportion of patients with atrial fibrillation that you see also have at least one other chronic disease?   - 0-10% - 11-30% - 31-50% - 51-80% - >80%   For those patients with atrial fibrillation who have at least one other chronic health condition, can you identify the main healthcare provider for them?   - Yes - No   In consultation with patients with AF and other chronic long-term conditions (multi-morbidity) do you usually communicate directly with (please, select all that apply):  the patient  their family   - non-family caregiver |
| *Clinicians have different training, orientations and views about a patient's role in their care which results in different approaches when working with people with long term conditions. This survey has been developed by the AFFIRMO team to understand these views and approaches, and the support needs of clinicians.*   \| **How do you grade your confidence in…** \| **Not at all confident** \| **Not very confident** \| **Neutral** \| **Confident** \| **Very confident** \| \| --- \| --- \| --- \| --- \| --- \| --- \| \| Assessing the level of patient’s engagement \|  \|  \|  \|  \|  \| \| \| Generally supporting patient engagement \|  \|  \|  \|  \|  \| \| \| Motivating patients in following medical prescriptions \|  \|  \|  \|  \|  \| \| \| Informing patients about disease and treatments \|  \|  \|  \|  \|  \| \| \| Assessing patient's health literacy \|  \|  \|  \|  \|  \| \| \| Empathising with patients \|  \|  \|  \|  \|  \| \| \| Assessing and managing patients' emotions \|  \|  \|  \|  \|  \| \| \| Effectively communicating with patients and their families \|  \|  \|  \|  \|  \| \| \| Effectively relating to patients and their families \|  \|  \|  \|  \|  \| \|  \| **As a healthcare professional, how important is it to you that your patients with chronic conditions:** \| **Extremely important** \| **Important** \| **Somewhat important** \| **Not important** \| **Not applicable** \| \| --- \| --- \| --- \| --- \| --- \| --- \| \| Are able to take actions that will help prevent or minimise symptoms associated with their health condition \|  \|  \|  \| \|  \| \|  \| \| Are able to maintain lifestyle changes needed to manage their long-term condition \|  \|  \|  \| \|  \| \|  \| \| Understand which of their behaviours make their condition better and which ones make it worse \|  \|  \|  \| \|  \| \|  \| \| Can follow through on medical treatments they need to do at home \|  \|  \|  \| \|  \| \|  \| \| Know what each prescribed medication does \|  \|  \|  \| \|  \| \|  \| \| Bring a list of questions when they come to the clinic \|  \|  \|  \| \|  \| \|  \| \| Are able to determine when they need to go to see a medical professional for care versus when they can manage the problem on their own \|  \|  \|  \| \|  \| \|  \| \| Are able to work out solutions when new situations or problems arise with their health condition \|  \|  \|  \| \|  \| \|  \| \| Want to be involved as a full partner with you in making decisions about their care \|  \|  \|  \| \|  \| \|  \| \| Tell you concerns they have about their health even when you do not ask \|  \|  \|  \| \|  \| \|  \| \| Want to know what procedures or treatments they will receive and why before the treatments are performed \|  \|  \|  \| \|  \| \|  \| \| Understand the different medical treatment options available for their long-term condition \|  \|  \|  \| \|  \| \|  \| \| Look for trustworthy sources of information about their health and health choices such as on the web, news, or books \|  \|  \|  \| \|  \| \|  \| |
| Overall, in your opinion, what are the main needs of patients with AF and other chronic health conditions? Please, select all that apply (up to 3):   - avoid stroke - control AF symptoms - balance the benefit/risk ratio due to anticoagulant treatment - managing the other comorbidities - control possible interactions between anticoagulation and other ongoing treatments - avoid hospitalizations - Other (please specify) _____________________   What health-related outcomes do you think are most important for patients with AF and other chronic conditions? Please, select all that apply (up to 3):  improvement of quality of life  maintenance of independence in daily life  increase longevity  pain control/relief  have the least need for health care  maintenance of social and leisure activities  improvement of mental/emotional health  be able to work  avoid/prevent adverse events (e.g. accidental falls)  other: _______________________________  What are the main difficulties with managing the health of patients with AF and other chronic conditions? Please, select all that apply (up to 3):  Managing multiple health conditions  Dealing with polypharmacy  Dealing with possible drug-drug or drug-food interactions  Evaluating the benefit-risk ratio of each treatment  Managing patients with poor social support or no caregiver  Explaining the medical recommendations to patients and/or caregivers  Uncertainty regarding patient and/or caregiver understanding of the medical recommendations given  Convincing the patient/caregiver of the importance of following the medical recommendations  Reaching a satisfactory adherence with medical recommendations by the patient  Communication with the patient’s family doctor or with other specialists  Conflicting opinions of medical colleagues regarding ‘best’ management due to multi-morbidity  Other (please specify): _______________________________ |

**Supplementary Figure 1.** Number of participants recruited for the survey, divided by group and country.

**References list**

1. EuroQol G. EuroQol--a new facility for the measurement of health-related quality of life. Health Policy. 1990;16(3):199-208.

2. Gleason LJ, Benton EA, Alvarez-Nebreda ML, Weaver MJ, Harris MB, Javedan H. FRAIL Questionnaire Screening Tool and Short-Term Outcomes in Geriatric Fracture Patients. J Am Med Dir Assoc. 2017;18(12):1082-6.

3. Graffigna G, Barello S, Bonanomi A, Lozza E. Measuring patient engagement: development and psychometric properties of the Patient Health Engagement (PHE) Scale. Frontiers in Psychology. 2015;6.

4. Williams GC, Grow VM, Freedman ZR, Ryan RM, Deci EL. Motivational predictors of weight loss and weight-loss maintenance. Journal of personality and social psychology. 1996;70(1):115.

5. Horne R, Weinman J. Self-regulation and self-management in asthma: exploring the role of illness perceptions and treatment beliefs in explaining non-adherence to preventer medication. Psychology and Health. 2002;17(1):17-32.

6. Duke CC, Lynch WD, Smith B, Winstanley J. Validity of a New Patient Engagement Measure: The Altarum Consumer Engagement (ACE) Measure. Patient. 2015;8(6):559-68.

7. Bakas T, Champion V. Development and psychometric testing of the Bakas Caregiving Outcomes Scale. Nursing Research. 1999;48(5):250-9.

8. Barello S, Castiglioni C, Bonanomi A, Graffigna G. The Caregiving Health Engagement Scale (CHE-s): development and initial validation of a new questionnaire for measuring family caregiver engagement in healthcare. BMC Public Health. 2019;19(1):1562.
